# Quality of life among home healthcare patients in Saudi Arabia: household-based survey

**DOI:** 10.1186/s12955-019-1095-z

**Published:** 2019-01-25

**Authors:** Khaled Al-Surimi, Iman Al-harbi, Ashraf El-Metwally, Motasim Badri

**Affiliations:** 1 Department of Health Systems and Management, College of Public Health and Health Informatics, King Abdullah International Medical Research Center, King Saud ben Abdulaziz University for Health Sciences, Ministry of the National Guard Health Affairs, Riyadh, Kingdom of Saudi Arabia; 2 King Abdullah International Medical Research Center, Riyadh, Kingdom of Saudi Arabia; 3 Primary Care and Public Health Department, School of Public health, Imperial College London, Riyadh, UK; 4 Department of Epidemiology and Biostatistics, College of Public Health and Health Informatics, King Abdullah International Medical Research Center, King Saud ben Abdulaziz University for Health Sciences, Ministry of the National Guard Health Affairs, Riyadh, Kingdom of Saudi Arabia

**Keywords:** Quality of life (QOL), Home health care, Saudi Arabia, WHOQOL

## Abstract

**Background:**

The need for home healthcare programs is an increasingly becoming important common component of healthcare worldwide, as an alternative to hospitalization, owing to the growing elderly population, chronic and acute diseases that need continuous monitoring and care. The overall aim of this study was to describe and assess the quality of life (QOL) and associated determinants among patients enrolled in the Home Health Care (HHC) program affiliated with the Ministry of National Guard Health Affairs in Riyadh, Saudi Arabia.

**Methods:**

This cross-sectional study was conducted among patients enrolled at the HHC program. The World Health Organization QOL questionnaire (WHOQOL-BREF) was used to collect data about the different domains of patients’ QOL. Logistic regression models were fitted to determine factors associated with QOL low score.

**Results:**

The study included 253 patients. Mean age was 67.05 (± 20.0)**.** The overall QOL for HHC patients was significantly affected by both socio-demographic and morbid characteristics. In the final Multivariate logistic regression models, marital status, and having psychological problems, stroke and number illness were independently associated with the overall QOL of HHC patients (*p* = .022, *p* = .002, *p* = .031, .057 respectively). The physical health domain score was significantly associated with education level, having psychological problems and stroke (*p* = .028, *p* = .002, *p* = .007 retrospectively) whereas the psychological domain score was significantly associated with age (*p* = < 0.001) and three types of chronic diseases: pulmonary (*p* = .002), psychological problems (*p* = < 0.001). The social domain score was significantly associated only with the marital status (*p* = .026). The environmental domain was significantly associated with the education level and having stroke (*p* = .017 vs .027).

**Conclusions:**

The overall QOL and its domains are significantly associated with several different factors. Many of these factors can be monitored and enhanced by improving quality of HHC services, thus improving the QOL of patients.

## Background

Home Health Care (HHC) consists of healthcare services that are provided to patients in their homes by qualified healthcare providers under the supervision of a physician. The purpose of these home healthcare services is to help improve all aspects of the quality of life (QOL) of patients, support their independence, and increase their level of well-being [1]. Globally, the need for such Health Home Care (HHC) programs has increased because of the increase in chronic and acute diseases that need continuous monitoring as well as the increase in the elderly population in which about 70.5% of HHC patients are 65 years and above [2].

The World Health Organization (WHO) defines QOL as an individual perception of their position in life in the context of the culture and value systems in which they live and in relation to their goals, expectations, standards, and concerns [3]. QOL is a broad and complex concept, hence, maintaining a high QOL and a high level of HHC services is challenging. Because care in the home environment is usually less controlled than care in a health facility, it involves more health-related hazards. It also includes a wide range of care providers such as unregulated workers, family members, and caregivers in a setting designed for daily living, not for providing regulated healthcare [4].

The majority of previous studies have focused on assessing a patient’s QOL within hospital settings rather than outside settings like HHC [4]. A study conducted in the USA assessed the QOL of 100 HHC patients using the WHO QOL (WHOQOL-100) questionnaire. The results showed a considerable improvement in clinical outcomes as well as the physical, psychological, and environmental domains of QOL after a patient became involved in a HHC program. [2]. In Brazil, a study measured the QOL among elderly patients receiving HHC using the WHOQOL-100 questionnaire. The results showed that patients had a good overall QOL although they had difficulties in some aspects such as a limited ability to perform daily activities [5] . Another study conducted in China aimed to assess the effect of HHC visits by nurses and monitoring patients’ condition through telephone calls on the QOL of HIV-infected heroin users. The results showed that all four domains of QOL significantly improved after HHC visits and ultimately resulted in better clinical outcomes, increasing the patient’s satisfaction and motivation to live [6]. A one-year longitudinal study conducted in Austria examined the effectiveness of a geriatric psychiatry home treatment program on the QOL of geriatric patients with mental problems. The brief WHO QOL (WHOQOL-BREF) questionnaire was used to measure the QOL. The results showed significant improvement in both the physical and psychological domains compared to the time of discharge, while social and environmental domains remained unchanged [7]. A study in Germany examined the effect of educational nurse-led home visits on QOL and functional status using the WHOQOL-BREF. The study results showed no significant positive effect due to educational nurse-led home visits on QOLF and functional status [8]. However, Courtney et al. (2009) reported that nurse-led home visit intervention, that consists of individualized exercise program and long-term telephone follow-u, had significant positive effects on QOL in their randomized controlled trial showing higher mean scores among interventional group compared with the control group [9].

Researchers have started to study and assess different aspects of HHC on QOL. For example, in Sweden they tried assessing the effect of patient-centered HHC services on the QOL of elderly patients. The results showed that more patient involvement in HHC plans and decisions improve their satisfaction and ultimately improve their QOL [10]. Another study in the USA measured the effect of home hemodialysis on QOL and found a significant increase in the physical domain score [11].

In Saudi Arabia, all the current HHC programs are public funded programs. The first HHC program was established in 1991 by the King Faisal Specialized Hospital, and was only for terminal cancer patients. Later in 1995, the Ministry of National Guard Health Affairs established its HHC program to provide home healthcare services to minimize the length of hospital stay. In 2008, the Saudi Ministry of Health started introducing the national HHC program [12]. This national HHC program, as cited in a study conducted by Almoajel et al. (2016), aims to “provide health services for all those who are in need of them, wherever they may be; in an endeavor to alleviate the suffering of waiting in hospitals or moving to get the service”, and to be provided according to the best practice of the international standards and within the framework of Islamic values and traditions of the Saudi society [12]. However, studies assessing the quality of home healthcare programs and the effect of such programs on a patient’s QOL within the Saudi Arabia healthcare context are very limited. For example, only one study conducted in Riyadh had assessed the impact of HHC programs located in five different governmental hospitals. The results showed that 86.5% of team members stated that there was a noticeable improvement in their patients’ condition after HHC visits [12]. Nonetheless, no previous studies have assessed the QOL of HHC patients; thus this study set out primarily to describe and assess the quality of life (QOL) and identifies its determinants among participants in the Home Health Care (HHC) program run by the Ministry National Guard Health Affairs in Riyadh, Saudi Arabia. It is hoped that the results will provide an empirical evidence-base for measuring and improving the quality of the HHC program in the long term in Saudi Arabia and in similar HHC program contexts in the region.

## Methods

### Study design and setting

This study is a cross-sectional study, using the Arabic version of WHOQOL-BREF. The study was conducted within the HHC program of National Guard Health Affairs in King Abdul-Aziz Medical City in Riyadh.

### Study population and sample

All patients enrolled in the HHC program who were available during the home visit and willing to participate in the study survey were included. The entire population of 300 patients enrolled in HHC program was surveyed. We recruited the study participants by visiting them at their home and conduct a face-to-face interview by well-trained social worker who is familiar with and working at HHC program of Ministry National Guard Health Affairs.

### Data collection

Data were collected using the WHOQOL-BREF questionnaire Arabic version for measuring QOL [13]. It is one of the best validated instruments for measuring QOL a cross-culturally generic instrument [14, 15]. It contains 26 items and covers four different domains of QOL: physical (seven items), psychological (six items), and social (three items), and environmental (three items). Two items about general health are also asked. Each item of the WHOQOL-BREF questionnaire is scored from 1 to 5 on a response scale, and then transformed to a 0–100 scale. The physical domain covers pain, energy, sleep, and daily activities. The psychological domain measures positive and negative feelings, ways of thinking, self-esteem, and body-image. The social domain covers questions about personal relationships and social support. The environmental domain contains questions about financial status, safety, home environment, and transportation [16]. The instrument reliability was measured using Cronbach’s alpha: the overall reliability for all questions on the WHOQOL-BREF was 0.91. For the physical health, social relationships, environmental, and psychological well-being domains, the reliabilities were 0.89, 0.81, 0.67, and 0.61. These findings show that the instrument has a good internal consistency and the study findings are reliable. Data were collected in 2016 using the face-to face interview approach conducted by a well-trained social worker who is familiar with and working at HHC program of Ministry National Guard Health Affairs in Riyadh.

### Data management and statistical analysis plan

All gathered data were entered into a computer using IBMSPSS (version 20, Oklahoma, US). Collected data were checked and cleaned to remove any entry errors. Data were summarized using descriptive statistics such as percentage and mean (±SD) and compared using inferential statistics such as the t-test and chi-square; as appropriate. For each domain the mean score was calculated by dividing the total score of all items in the domain by the number of items in the domain. Then the overall mean score had been calculated by adding up the mean score for each domain divided by number of the domains. For the purpose of regression analysis, subjects were divided into a high-score group and low-score group based on the median value as cut of point of the total WHOQOL-BREF score and four domain scores as done in similar published studies [17, 18]. Survey items associations with the QOL were examined using logistic regression analyses in which QOL score (high versus low) was the dependent variable. Variables found significant in univariate logistic regression (< 0.05) were included in the final multivariate logistic regression model analysis. All tests were two-sided and a *p* < 0.05 was considered significant. Missing data were imputed using the multiple imputation technique [19].

## Results

### Study respondents’ characteristics

The respondents’ characteristics are shown in Table 1. About two thirds (63%) of the respondents were female. The average age was 67.05 (± 20.0), where the majority (73.3%) were above 60 years old and only about 12% were less than 40 years old. About two thirds (61.3%) of the respondents were illiterate and only 38.719% can read and write and above. More than half (59.7%) of the respondents were married. The majority (85%) were unemployed and only 15% had jobs. The majority of respondents (86.2%) lived in shared houses and only 13.8% had an independent house. Regarding type of morbid conditions, 60.1% of them had hypertension, 60.5% had diabetes, 15.4% had cancer, 28.5% had arthritis, 11.5% had psychological problems**, 0**8.7% had pulmonary disease, and 26.9% had stroke.

**Table 1:** Study respondent characteristics, (*N = 253*)

Characteristic	Number of participants	%
Age (Years) 67.05 ± 20.0		
Gender		
Female	160	63.2
Male	93	36.8
Education level		
Illiterate	155	61.3
Literate	98	38.7
Marital status		
Single	102	40.3
Married	151	59.7
Employment status		
Unemployed	215	85.0
Employed	38	15.0
Housing type		
Independent	35	13.8
Shared	218	86.2
Chronic disease		
Cardiac disease		
No	236	93.3
Yes	017	06.7
Hypertension		
No	101	39.9
Yes	152	60.1
Arthritis		
No	181	71.5
Yes	072	28.5
Cancer		
No	214	84.6
Yes	039	15.4
Pulmonary disease		
No	231	91.3
Yes	022	08.7
Diabetes		
No	100	39.5
Yes	153	60.5
Psychological problems		
No	224	88.5
Yes	029	11.5
Stroke		
No	185	73.1
Yes	068	26.9

### HHC patients’ quality of life (QOL)

The QOL domains scores (mean and standard deviation) are shown in Table 2. Overall, the total mean score for all four QOL domains was 3.78 ± 0.78. For the physical health, social relationships, environmental, and psychological well-being domains, the mean scores were 2.22 ± 0.85, 3.37 ± 0.65, 3.37 ± 0.68, and 2.87 ± 0.78, respectively. Among the physical health items, participants’ satisfaction about the quality of their sleep had the highest mean score (3.19 ± 0.86), and their ‘satisfaction about the quality of their sex life was the highest (4.05 ± 0.60) among the social domain items. The majority of participants were satisfied with the conditions of their living space (4.35 ± 0.60) reflecting the quality of environmental domain. On the other hand, although the majority of the participants were satisfied with themselves (3.42 ± 0.69), a large percentage expressed that they had negative feelings very often (3.34 ± 0.92) among psychological items.

**Table 2:** QOL domain scores for HHC patients (*N = 253)*

Domain	No.	Mean	SD
Physical health domain			
To what extent do you feel that physical pain prevents you from doing what you need to do?	253	2.17	0.99
Do you have enough energy for everyday life?	253	1.89	0.81
How satisfied are you with your sleep?	253	3.19	0.86
How well are you able to get around?	253	1.67	0.74
How satisfied are you with your ability to perform your daily living activities?	253	2.31	0.90
How much do you need any medical treatment to function in your daily life	253	1.94	0.72
How satisfied are you with your capacity for work?	253	2.35	0.92
TOTAL Score		2.22	0.85
Social relationships domain			
How satisfied are you with your personal relationships?	253	3.76	0.69
How satisfied are you with the support you get from your friends?	253	3.53	0.66
How satisfied are you with your sex life?	253	4.05	0.60
TOTAL Score		3.78	0.65
Environmental domain			
How safe do you feel in your daily life?	253	3.26	0.84
How satisfied are you with the conditions of your living place?	253	4.35	0.60
Have you enough money to meet your needs?	253	3.17	0.52
How satisfied are you with your access to health services?	253	3.97	0.65
How available to you is the information that you need in your day-to-day life?	253	2.95	0.58
To what extent do you have the opportunity for leisure activities?	253	1.66	0.82
How healthy is your physical environment	253	3.6	0.70
How satisfied are you with your transport?	253	4	0.70
TOTAL Score		3.37	0.68
Psychological domain			
How much do you enjoy life?	253	2.44	0.75
How well are you able to concentrate?	253	2.25	0.92
How satisfied are you with yourself?	253	3.42	0.69
Are you able to accept your bodily appearance?	253	2.7	0.59
How often do you have negative feelings such as blue mood, despair, anxiety, depression?	253	3.34	0.92
To what extent do you feel your life to be meaningful?	253	3.06	0.82
TOTAL Score		2.87	0.78

### HHC patients’ quality of life (QOL) by socio-demographic characteristics

The overall QOL score differed significantly in the two groups in terms of marital status (*p* = 0.002), Employment status (*p* = 0.019) hypertension (*p* = 0.040), and having psychological problems (*p* < 0.001) and stroke (*p* = 0.003). However, in the physical domain, education level, employment status, housing type, and having cancer psychological problems and stroke differed significantly in the two groups. Regarding the psychological domain, significant differences were observed in age, education level, marital status, and having hypertension pulmonary disease, psychological problems and stroke. In the social relationships domain, the only significant differences were related to marital status, and having psychological problems. As for the environmental domain, there was significant difference in age, education level, employment status, housing type, and having diabetes and stroke see the details in the additional file 1).

### Factors associated with patients’ overall quality of life (QOL) and its domains

In logistic regression analysis factors significantly associated with overall score of QOL for HHC patients were marital status (OR = .516, 95% CI [.293, .910] *p* = .022,), psychological problems (OR = 5.95, 95% CI [1.98, 17.93], *p* = .002) and stroke (OR = 2.10, 95% CI [1.07, 4.11], *p* = .031) while the number of illnesses has a borderline significance on overall QOL (OR = 1.47, 95% CI [0.99, 2.20], *p* = .057,). However, in the physical domain significant factors were education, psychological problems, and stroke. Whereas in the psychological domain significant factors were age, and having psychological problems, pulmonary disease and stroke). In the social relationships domain, the only one significant factor was marital status. In the environmental domain, the significant factors were education, and having stroke, Table 3.

**Table 3:** Logistic regression analysis for factors associated with the overall QOL and its sub-domains

Variable	Univariate		Multivariate	
	OR [95% CI]	*p*	OR [95% CI]	*p*
OVERALL QUALITY OF LIFE (ALL DOMAINS)				
Marital status:				
*Single*	1	–	1	–
*Married*	0.44[0.26–0.74]	.002	.516 [.293–.910]	.**022**
Disease name:				
Hypertension	1.73 [1.04–2.88]	.034	1.27[.56–2.90]	.567
Psychological problems	5.25 [1.93–14.25]	.001	5.95[1.98–17.93]	**.002**
CVA/Stroke	2.46 [1.37–4.41]	.003	2.10 [1.07–4.11]	**.031**
Number of illnesses	1.72 [1.34–2.21]	.000	1.47[.99–2.20]	.057
PHYSICAL HEALTH DOMAIN				
Education level:				
Literate	1	–	1	–
Illiterate	1.82 [1.09–3.03]	.029	1.92 [1.07–3.44]	.**028**
Disease name:				
Psychological problems	2.58 [1.10–6.07]	.030	4.53 [1.78–11.55]	**.002**
Stroke	2.76 [1.52–5.02]	.001	2.51[1.29–4.88]	.**007**
Number of illnesses	1.50 [1.178–1.91]	.001	1.23[0.93–1.61]	.161
PSYCHOLOGICAL WELL-BEING DOMAIN				
Age in years	1.02 [1.01–1.03]	.004	1.04[1.02–1.05]	**< .001**
Marital status:				
Single	1	–	1	–
Married	0.48[0.29–0.080]	.005	0.60, [0.33–1.09]	.095
Disease name:				
Hypertension	1.76 [1.06–2.92]	.030	1.75[0.70–4.35]	.230
Pulmonary disease	2.79[1.05–7.37]	.039	6.20[1.90–20.24]	**.002**
Psychological problems	4.27[1.67–10.88]	.002	23.47[6.05–91.12]	**<.001**
Stroke	2.57[1.43–4.61]	.002	2.98 [1.44–6.15]	**.003**
Number of illnesses	1.59 [1.25–2.04]	.000	0.98[0.64–1.51]	.939
SOCIAL RELATIONSHIPS DOMAIN				
Marital status:				
Single	1	–	1	–
Married	0.49[0.30–0.82]	.007	0.55[0.33–0.93]	**.026**
Disease name:				
Psychological problems	2.77[1.18–6.56]	.020	2.27[.94–5.45]	.068
ENVIRONMENTAL DOMAIN				
Age in years	1.02 [1.01–1.03]	.004	1.00[.99–1.02]	.840
Education level:				
Literate	1	–	1	–
Illiterate	2.39 [1.41–3.99]	.001	1.97 [1.13–3.746]	**.017**
Disease name:				
Diabetes	1.69[1.01–2.83]	.045	0.66 [0.35–1.69]	.515
Stroke	3.14[1.65–5.96]	.000	2.21 [1.10–4.40]	**.027**
Number of illnesses	1.51[1.18–1.93]	.001	1.37[0.93–2.00]	.108

Boldface figures show the only significant variables in final model of multivariate analysis

## Discussions

The aim of this study was to assess the QOL for homecare patients and identify different associated factors with QOL among patients of the Ministry National Guard HHC program. Although studies on the QOL of HHC patients and the effect of such programs on QOL are very limited, previous studies showed that having HHC services were effective in improving all domains of QOL [6]. The majority of our study participants were on average above 67.05 years old showing that elderly patients usually have more chronic diseases and need more HHC services [5]. This is consistent with previous studies, which found that the majority of HHC patients are usually older than 60 years old [2, 4, 9].

### HHC patients’ quality of life (QOL) domains

The results of QOL domain scores show that in the physical health domain aspects, participants were mostly satisfied with the quality of their sleep, while a study about HHC and QOL in cancer patients in Turkey showed that sleep disorders were a major concern among HHC patients. The differences in results could be due to the severity of the patients’ conditions because HHC services in Turkey are only provided for patients who are in the end stages of disease with very serious disabilities [20] while in our study we included all chronic patients enrolled in the HHC program. In the social domain aspects, participants’ satisfaction about the quality of their sex life was the highest, while they scored low in the physical domain with respect to having enough energy for everyday activities. A study done in Turkey measured the effect of HHC on QOL in patients diagnosed with gastrointestinal cancer and showed that almost all of their participants suffered from lack of energy, which is in line with our results, while they also had decreased sexual interest, which contradicts our results. This might be due to the chronic condition of the participants since cancer is a leading cause of different types of disabilities [21] including negative affect on their sex life. Besides, another possible explanation might be attributed to the conservative culture that doesn’t prefer revealing publicly negative opinion about such sensitive personal and family life issues. In the environmental domain aspects, participants were mostly satisfied with the conditions of their living place, while in the psychological domain, the majority of the participants were satisfied with themselves, but also a large percentage expressed that they very often had negative feelings. One of the possible interpretations of the participants being satisfied about the conditions of their living place is that most these houses are sponsored by Ministry of National Guard Health Affairs, as part of the employee benefits package. At the level of total scores, our study showed that HHC patients are more satisfied with their quality of social relationships and environmental domain and less satisfied with the quality of their physical health and psychological aspects of life. These high quality of life scores for social relationships and environment domains are in alignment with home health care study conducted in Turkey, comparing patients who live in their homes with those living in an assisted living facility [20]. On the other hand, our study findings differ from study findings on quality of life (QOL) among home health care patients in United States. This study showed that the lowest mean score was for the social domain and the highest was for physical domain [2]. This discrepancy could be generally due to the differences in the social culture and healthcare settings of the two studies; where in Saudi context patient usually should live within the family as part of the local culture where they might get more social support.

### Factors associated with QOL for HHC patients

The results of multivariate regression analysis showed that being married and having had a stroke have significant effects on the overall QOL. Our results also showed that having psychological problems was a significant factors associate with the overall QOL. This is consistent with the findings of a study that assessed the QOL of patients with dementia receiving HHC in European countries, in which the results showed that having depressive symptoms significantly lowered the overall QOL [22]. Our results showed no association between age, housing type, or educational level with the overall QOL, while a study assessed the overall QOL among older HHC clients in Czech Republic found that younger, educated participants and those living in shared houses had a higher overall QOL compared to others. These inconsistent results could be due to the differences in the demographic and clinical characteristics of the study participants. The Czech study also showed that morbid conditions were significantly associated with having a lower overall QOL; this result is fairly consistent with our result, which showed a borderline association between the number of illnesses the patients had and their overall QOL [23].

At the QOL domains level, the QOL in the physical health domain was affected by three factors, the level of education, having psychological problems, and having suffered from a stroke. A study in China reported the effects of nurse-delivered home visits combined with telephone calls on medication adherence on QOL showed that a high education level is associated with good medication adherence, which then improves the physical QOL, which is consistent with our study findings [6].

In the psychological health domain, the most significant predictors for QOL were age and whether the patient has any psychological problems, followed by having pulmonary disease or having suffered a stroke. This significant effect of the age on the psychological QOL in our study contradicts the findings of a study done in the USA and South Korea showed that among the predictors of QOL is association between age and physical health domain not the psychological health domain [2, 24]. These contradicted results might be due to not only the differences in participants’ characteristics but also the culture differences. In Saudi culture, the elderly people usually live within the family where they expect more emotional support from the family members in which might affect their psychological health rather than physical health. Our results also found an association between psychological QOL and having pulmonary disease. This result is almost consistent with the results of a study that measured the QOL in elderly nursing home residents with chronic respiratory diseases: the results showed an association between the psychological domain and frequent coughing [18].

Our study results showed being married had a significant association with the quality of social aspects domain. The study participants scored high when it came to their satisfaction with personal relationships, and this is in line with the results of a study done in the USA that showed a positive relationship between the number of people available for support and the emotional and social QOL [24]. On the other hand, our results showed no association between age, housing type, or educational level with the social aspects domain. In the environmental domain, the main predictors were stroke followed by the number of illnesses of the patient.

In brief, our study and the previous results confirmed that having psychological problems or stroke are the most important factors associated with QOL of home healthcare patients, where the age had shown a major effect on the psychological well-being of the participants [17, 22, 23].

### Study limitations and strengths

To the best of our knowledge, this is the first study in Saudi Arabia to assess the QOL of HHC patients, which, also considering the lack of prior research studies on the same topic globally, is strength of the study. There is a great opportunity for future studies to build on the basis of the results and evidence provided by this study. Because it was conducted in one setting, the study results may not reflect the general conditions of all HHC patients in the county. In contrast, it represents only those involved in the Ministry of National Guard HHC program. Although the WHOQOL-BREF questionnaire is a valid and reliable instrument, it has been stated that its assessment of the social domain QOL is not as high reliable as other domains [14]. However, this might social desirability the results related to social domain results this study. Finally, this was a cross-sectional study in one site, which might limit scope the generalizability of findings. Thus, we recommend in future studies to include more than HHC program to avoid the limitations of this study’s design. Because QOL is a broad concept and studies on this topic are rare in Middle East, further studies are needed to gain more knowledge and insight into the different factors affecting QOL and ways to control them to improve QOL. This will help provide better QOL.

### Conclusions and implications

This study used the WHOQOL-BREF to assess the four main domains of QOL (physical, psychological, social, and environmental) of HHC patients. We conclude that QOL domains are affected by many different factors, and our results show that some factors affect more than one domain (e.g., psychological problems and stroke). Dealing with those factors and understanding their effect on QOL is a shared responsibility of healthcare providers, HHC program planner s and family caregivers; many of these factors can be monitored and enhanced by improving the quality of HHC services and thus improve the QOL of patients. The National Guard HHC program can be improved to provide high-quality services by well-trained teams.

Our findings suggest that there are opportunities for improvement initiatives for quality of life among HHC patients. The priority of such initiatives should focus on the psychological well-being, especially patients with stroke. It’s also worth mentioning that although the quality of social relationships of HHC patients seems relatively good, showing that the local social culture accept patient to live as valuable member of the family and deserves family’s members support, this social culture should be maintained and encouraged by healthcare providers and those in charge of HHC program. Moreover, quality of life among HHC patients could be improved by enhancing the communication skills of HHC providers with care recipients and family caregivers; this could foster both physical and psychological health aspects as well as prompting the self-determination and person-centered care thinking among HHC patients, which requires involving them in HHC plans and decisions as strategy to improve patients satisfaction and ultimately their QOL [10]. Future research should identify and assess interventions that are targeted to improving the quality of life in HHC patients.

## Abbreviations

HHC: Home Health care
QOL: Quality of life
WHO: World Health Organization
